# Features of Emergency Medical System calls that facilitate or inhibit Emergency Medical Dispatcher recognition that a patient is in, or at imminent risk of, cardiac arrest: A systematic mixed studies review

**DOI:** 10.1016/j.resplu.2021.100173

**Published:** 2021-11-18

**Authors:** Kim Kirby, Sarah Voss, Emma Bird, Jonathan Benger

**Affiliations:** a South Western Ambulance Service NHS Foundation Trust, Eagle Way, Exeter EX2 7HY, United Kingdom; b University of the West of England, Blackberry Hill, Stapleton, Bristol BS16 1DD, United Kingdom

**Keywords:** Emergency Medical Service, Out-of-hospital cardiac arrest, Emergency medical dispatch

## Abstract

**Aim:**

To identify and appraise evidence relating to the features of an Emergency Medicine System call interaction that enable, or inhibit, an Emergency Medical Dispatcher’s recognition that a patient is in out-of-hospital cardiac arrest, or at imminent risk of out-of-hospital cardiac arrest.

**Methods:**

All study designs were eligible for inclusion. Data sources included Medline, BNI, CINAHL, EMBASE, PubMed, Cochrane Database of Systematic Reviews, AMED and OpenGrey. Stakeholder resources were screened and experts in resuscitation were asked to review the studies identified. Studies were appraised using the Mixed Methods Appraisal Tool. Synthesis was completed using a segregated mixed research synthesis approach.

**Results:**

Thirty-two studies were included in the review. Three main themes were identified: Key features of the Emergency Medical Service call interaction; Managing the Emergency Medical Service call; Emotional distress.

**Conclusion:**

A dominant finding is the difficulty in recognising abnormal/agonal breathing during the Emergency Medical Service call. The interaction between the caller and the Emergency Medical Dispatcher is critical in the recognition of patients who suffer an out-of-hospital cardiac arrest. Emergency Medical Dispatchers adapt their approach to the Emergency Medical Service call, and regular training for Emergency Medical Dispatchers is recommended to optimise out-of-hospital cardiac arrest recognition. Further research is required with a focus on the Emergency Medical Service call interaction of patients who are alive at the time of the Emergency Medical Service call and who later deteriorate into OHCA.

PROSPERO registration: CRD42019155458.

## Introduction

Out-of-hospital cardiac arrest (OHCA) is a catastrophic event requiring immediate intervention if a patient is to have any chance of survival. Survival to hospital discharge following OHCA is poor and varies globally with 11.7% of patients surviving to hospital discharge in Europe compared to 4.5% of patients in Asia.^1^ When an Emergency Medical Service (EMS) call is received regarding a patient who is in OHCA or at imminent risk of OHCA a crucial factor in the patient's survival is the recognition of the severity of the patient's condition. Early recognition by an Emergency Medical Dispatcher (EMD) that a patient is critically unwell instigates the rapid dispatch of EMS. Grading of EMS calls is an important part of the “Chain of Survival” in OHCA^2^ and in 2005 the Chain of Survival was revised to acknowledge the importance of recognising critical illness and/or acute coronary syndrome and cardiac arrest prevention, both in and out of hospital.^3^ When a patient suffers an OHCA the initial minutes following collapse are critical.^4^ Each second without resuscitation decreases that patient’s chances of survival.^5^ Early intervention by bystanders, guided by EMDs, is imperative and quality CPR and bystander defibrillation are dependent on the EMD or bystander recognising that the patient is in OHCA.^6^

Deakin^7^ demonstrated that all links in the chain of survival are not equal in terms of the numbers progressing through each stage. Improving the first link in the chain of survival - early recognition and call for help - has the potential to have the largest impact on OHCA patients due to the comparative volume of patients at this stage. Recognition, during the EMS call, of patients who are at imminent risk of OHCA will ensure that EMS staff arrive as quickly as possible to either treat the cardiac arrest as soon as it occurs or, better still, prevent it from happening through the provision of early treatment.^8^

The International Liaison Committee on Resuscitation (ILCOR)^9^ recognise studies which address knowledge gaps associated with OHCA recognition to be both high impact and high priority. ILCOR note that an area that requires further research is the optimal questions and instructional sequences to provide to callers to enhance recognition of OHCA and provision of CPR. Other systematic reviews have been completed in this area. Drennan et al.^10^ reviewed quantitative papers concerning patients presumed to be in OHCA. The authors evaluated the diagnostic accuracy of dispatch centres to diagnose OHCA and investigated EMS call characteristics that impact on the ability of EMDs to diagnose OHCA. Findings indicated variance in the sensitivity and specificity of OHCA recognition across dispatch centres with no difference in accuracy between dispatch criteria/algorithm or with the level of education of the EMDs. Vaillancourt and colleagues^11^ aimed to determine whether description of specific symptoms by the caller improved the accuracy of the identification of OHCA by systematically reviewing interventional and observational studies. Findings indicated the importance of enquiry regarding consciousness and breathing to determine OHCA. In addition, the review highlighted that abnormal breathing is a significant barrier to recognition of OHCA and the presence of seizures can be an indication of OHCA.

This systematic mixed studies review (SMSR) aimed to appraise evidence that investigates the features of an EMS call that facilitate or inhibit recognition by the EMD that a patient is in cardiac arrest, or at imminent risk of OHCA.

## Methods

### Protocol and registration

The protocol for this systematic review was registered on International Prospective Register of Systematic Reviews (PROSPERO), registration number: CRD42019155458 and can be accessed on https://www.crd.york.ac.uk/prospero/.

The protocol was registered on 5th November 2019.

### Identification of studies

The search terms used in the SMSR were developed with a Clinical Research Librarian and reviewed amongst the authorship team. The search terms were developed using MeSh Headings where relevant and combined using Boolean Operators. The initial searches were performed between November and December 2019 and rerun in May 2021. The final MEDLINE search strategy developed is shown in appendix one.

### Information sources

The following databases were searched by KK: Medline, BNI, CINAHL, EMBASE, PubMed, Cochrane Database of Systematic Reviews, AMED, OpenGrey. Stakeholder resources were also searched by KK and included: International Liaison Committee on Resuscitation, International Academies of Emergency Dispatch and NHS England. Three international resuscitation experts, with an interest in Emergency Medical Service dispatch, were identified to review the results of the systematic literature searches and provide expert opinion on any relevant additional resources that were not already identified during the search process. Any eligible literature was hand searched to ensure all relevant backward citations were identified from the papers.

### Inclusion criteria

Study Design: Primary quantitative, qualitative and mixed methods research.

Types of participants: Studies investigating adults and children who are in out-of-hospital cardiac arrest, or at imminent risk of out-of-hospital cardiac arrest.

Types of outcomes: Studies investigating the features of an EMD/caller interaction that facilitate or inhibit recognition by the EMD that a patient is in out-of-hospital cardiac arrest, or at imminent risk of out-of-hospital cardiac arrest.

Date of publication: 1990 to May 2021.

Country: No restrictions were applied.

Language: Published in the English language.

Grey Literature: Included

Study selection and categorisation

Eligibility criteria were applied to the search results and studies identified in the searches were imported to Covidence literature screening software (Veritas Health Innovation, Melbourne, Australia). Title and abstract screening were completed by the first reviewer (KK) with a validation sample of 20% independently screened by a second reviewer (SV). This process was repeated when reviewing the full texts. There was an ongoing dialogue between the reviewers to resolve any uncertainties, and there was no disagreement between reviewers regarding the validation sample. The categorisation phase involved determining whether the papers were qualitative, quantitative, or mixed methods. The studies were split into the five types of study described in the Mixed Methods Appraisal Tool (MMAT).^12^ The decision to categorise the studies in this way was a pragmatic one based on an intention to use the MMAT to assess the quality of included studies.

### Data extraction

Data were extracted which addressed the features of the EMS call that enable, or inhibit, an Emergency Medical Dispatcher’s recognition that a patient is in OHCA, or at imminent risk of OHCA. The first reviewer (KK) extracted data from the categorised studies into a table of findings and into an Excel spreadsheet. The second reviewer (SV) independently validated 20% of data extraction with no disagreement.

### Planned methods of analysis

This SMSR set out to synthesise data and results produced from studies with diverse designs to include quantitative, qualitative and mixed methods designs13., 14.. A segregated mixed research synthesis approach as described by Sandelowski et al.^15^ was the underlying method used to integrate the findings from both qualitative and quantitative research studies. The two mixed methods study identified during the search phase were fractionated, as described by Frantzen and Fetters,^16^ into qualitative and quantitative data. The segregated design recognises the distinct differences between qualitative and quantitative research. The approach requires separate analysis of the quantitative and qualitative findings before synthesising into a set of conclusions. Quantitative and qualitative data were coded in Excel before synthesis into themes. This segregated design is appropriate for use in the context of this SMSR because the research found during the literature search was complementing rather than confirming, or refuting. The mixed research synthesis was defined as the configuration rather than the assimilation of research findings as described in Sandelowski et al.’s work.15., 17.

### Quality assessment

The Mixed Methods Appraisal Tool (MMAT)^12^ has been designed specifically for mixed research synthesis. The MMAT allows the critical appraisal of five types of studies, to include: qualitative research; randomised controlled trials; non-randomised studies; quantitative descriptive studies; mixed methods studies. Originally developed in 2006,^13^ the tool was revised in 2011^18^ The current version was further revised following a Delphi study, interviews with MMAT users and a literature review of critical appraisal tools.^12^

Each paper was scored using the MMAT. Quality scores were calculated by grading the papers from 0% to 100% based on the quality criteria met. The papers scored 20% for each of the quality criteria met and grading was completed by KK with 20% of the sample validated by SV, with no disagreement. This type of scoring using the MMAT has been used previously.19., 20., 21., 22. Papers scoring above 80% were graded as high certainty, scores of 80% were graded as moderate certainty and below 80% as low certainty. As recommended by Hong et al.^23^ the context of individual scoring is included in the limitation sections of the certainty tables (supplementary Tables S3-S9).

## Results

Thirty-two studies were included in the final review. The study flow diagram is shown in Appendix B and Table 1 details the study characteristics. These 32 studies were categorised using the MMAT categories^23^ and are shown in their categories in supplementary Table S1. We set out to include all studies that investigated the features of an EMD/caller interaction for both patients already in cardiac arrest (“recognition” studies) and patients at risk of imminent cardiac arrest (“prediction” studies). Unfortunately no “prediction studies” met the inclusion criteria and investigated the features of the EMS call interaction for patients who were unequivocally alive (i.e. definitely not in cardiac arrest) at the time of the EMS call. “Recognition studies” therefore dominated this SMSR, and challenges associated with the recognition of cardiac arrest were apparent.

**Table 1:** Study Characteristics.

High certainty quantitative papers						
First Author	Date of data collection/publication	Country	Design	Number and types of participants	Main themes identified	Quality grade
Berdowski^24^	2004/2009	Netherlands	Prospective observational study	11,416 high priority emergency, non traumatic EMS calls	Key features of the EMS call interaction; Managing the emergency call; Patient colour	High
Meischke^25^	2013–2016/2017	United States	A parallel prospective randomised controlled trial	128 Emergency Medical Dispatchers	Managing the emergency call	High
Chien^26^	2015–2016/2019	Taiwan	Retrospective cross-sectional study	424 EMS calls for non-traumatic adult OHCA	Key features of the EMS call interaction; Emotional distress	High
Moderate certainty quantitative papers						
Castren^27^	1996/2001	Finland	Prospective study	328 EMS calls reporting non-traumatic OHCA that were witnessed or had bystander-initiated CPR ongoing.	Managing the emergency call; Emotional distress	Moderate
Garza^28^	2000/2003	US	Retrospective Review of EMS Dispatch Data	520 OHCA EMS calls	Managing the emergency call	Moderate
Nurmi^29^	1996/2006	Finland	Prospective Study	776 OHCA EMS calls	Key features of the EMS call interaction; Managing the emergency call	Moderate
Ma^30^	2004/2007	Tapei	Retrospective Observational Study	301 OHCA EMS calls	Key features of the EMS call interaction; Managing the emergency call	Moderate
Clawson^31^	2004–2006/2008	United Kingdom	Retrospective Comparative Study - before and after study	2.33 million EMS calls	Key features of the EMS call interaction; Managing the emergency call	Moderate
Roppolo^32^	Unclear/2009	United States	Prospective before and after study	962 OHCA patients	Key features of the EMS call interaction; Managing the emergency call	Moderate
Lewis^33^	2011/2013	United States	Retrospective cohort study	590 OHCA EMS calls	Key features of the EMS call interaction; Managing the emergency call	Moderate
Hardeland^34^	2007–2011/2014	Norway	Observational Study	414 OHCA patients	Key features of the EMS call interaction; Managing the emergency call	Moderate
Travers^35^	2012/2014	France	Prospective Observational Study	144 OHCA patients	Key features of the EMS call interaction; Managing the emergency call	Moderate
Moller^36^	2013/2016	Sweden	Observational Registry Study	930 OHCA patients	Key features of the EMS call interaction; Managing the emergency call	Moderate
Biancardi^37^	Unclear/2017	Malta	Simulation study	52 nurses	Key features of the EMS call interaction; Managing the emergency call	Moderate
Mirhaghi^38^	2015/2017	Iran	Content analysis OHCA emergency calls	80 OHCA EMS calls	Key features of the EMS call interaction; Managing the emergency call	Moderate
Hardeland^55^	2014/2017	Norway	Prospective, interventional study	561 OHCA calls	Key features of the EMS call interaction; Managing the emergency call	Moderate
Riou^39^	2014–2015/2018	Australia	Retrospective Linguistic Analysis	176 OHCA EMS calls	Key features of the EMS call interaction; Managing the emergency call	Moderate
Derkenne^40^	2012–2018/2020	France	Repeated cross-sectional study	321 OHCA EMS calls	Key features of the EMS call interaction; Managing the emergency call	Moderate
Mao^41^	2018/2020	Singapore	Prospective before and after study	513 EMS calls for unconscious patients	Key features of the EMS call interaction; Managing the emergency call	Moderate
Schwarzkoph^4^2	2014–2018/2020	United States	Retrospective cohort study	3502 OHCA EMS calls	Key features of the EMS call interaction; Managing the emergency call; Patient colour	Moderate
Stangenes^43^	Unclear/2020	United States	Analysis OHCA EMS calls	434 OHCA EMS calls	Managing the emergency call	Moderate
Tamminen^44^	2017/2020	Finland	Descriptive pilot study - retrospective registry study	80 OHCA EMS calls	Key features of the EMS call interaction; Managing the emergency call; Patient colour	Moderate
Gram^45^	2017–2020/2021	Denmark	A quality assessment study	673 OHCA EMS calls	Managing the emergency call	Moderate
Riou^56^	2014–2015,2021	Australia	Retrospective cohort study	422 OHCA EMS calls	Key features of the EMS call interaction; Managing the emergency call	Moderate
Low certainty quantitative papers						
Bang^46^	2000–2001/2003	Sweden	Prospective study	100 OHCA EMS calls	Key features of the EMS call interaction; Managing the emergency call; Emotional distress	Low
Bohm^47^	2004–2006/2009	Sweden	Before and after study	570 OHCS EMS calls	Key features of the EMS call interaction; Managing the emergency call	Low
High certainty qualitative papers						
Bang^48^	Unclear/2002	Sweden	Qualitative semi-structured interview study	10 Emergency Medical Dispatch staff	Managing the emergency call	High
Riou^49^	2014–2015/2018	Australia	Conversation Analysis	66 OHCA EMS calls	Managing the emergency call	High
Moderate certainty qualitative papers						
Jensen^50^	2009/2012	Canada	Qualitative telephone interview study using the Theory of Planned Behaviour	24 Ambulance Communication Officers	Key features of the EMS call interaction; Managing the emergency call	Moderate
Alfsen^51^	2021/2015	Denmark	Inductive thematic analysis EMS calls	21 OHCA EMS calls	Managing the emergency call; Emotional distress	Moderate
High certainty mixed methods papers						
Hardeland^52^	2013–2014/2016	Norway	Observational study, non-participant observation and in-depth interviews	1095 OHCA EMS calls, Non-participant observations at 3 Emergency Medical Communication Centres, 19 interviews with EMDs	Key features of the EMS call interaction; Managing the emergency call	High
Moderate certainty mixed methods papers						
Watkins^53^	2013–2014/2021	United Kingdom	Mixed methods retrospective study– qualitative call analysis and OHCA data analysis	39,136 EMS dispatches	Key features of the EMS call interaction; Managing the emergency call	Moderate

### Quality assessment

Supplementary Table S2 shows the grading of papers grouped into quantitative, qualitative and mixed methods studies.

### Overall synthesis

**Fig. 1:**
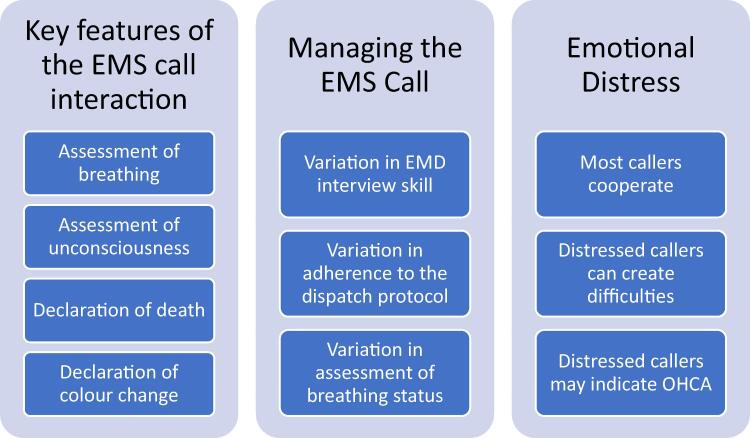
Mixed Methods Synthesis of Findings: Main Themes.

Fig. 1 displays the mixed methods synthesis of findings and is described further below. There were three main themes:

Key features of the EMS call interaction; Managing the emergency call; Emotional distress.

## Key features of the EMS call interaction

### Assessment of breathing

#### The recognition of abnormal/agonal breathing is critical in OHCA

Within the studies reviewed many had a focus on abnormal/agonal breathing for the reason that abnormal breathing, or respiratory distress, are indicators of OHCA.24., 29., 46., 50., 53., 54., 35. Tamminen et al.^44^ identified that ‘not breathing’ and ‘abnormal breathing’ are significant trigger phrases used to describe OHCA. Where breathing is adequately addressed on the EMS call an OHCA is more likely to be recognised.32., 33., 36., 40., 47., 35.

#### Abnormal/agonal breathing in out-of-hospital cardiac arrest is ambiguous and easy to misinterpret

Although the studies recognised the importance of recognising abnormal/agonal breathing a frequent reason for not recognising OHCA during the EMS call is the misinterpretation, or lack of clarity, regarding breathing status.26., 30., 33., 34., 37., 39., 41., 47., 52., 55. Assessment of breathing can be delayed in an OHCA presenting with seizure activity and in patients where an incorrect medical condition is described.42., 43. The addition of a question focused on regular breathing to the Medical Priority Dispatch System (MPDS) seizure protocol improved OHCA recognition.^31^ EMDs are reliant on the caller’s interpretation and communication of the situation48., 50. and EMDs describe trusting the caller’s description of breathing until proved inaccurate.^50^ However, EMDs also describe working with the descriptions provided by the witness, with some EMDs employing personalised intervention-based identification techniques in an attempt to identify abnormal breathing.^48^ Alfsen et al.^51^ noted that where a witness is near to the patient during the EMS call, they can better describe any abnormal breathing and assist the EMD with the recognition of OHCA.

### Assessment of unconsciousness

Watkins and colleagues^53^ found a description of unconsciousness to have high sensitivity and low specificity for OHCA and that assessing unconsciousness on the EMS call can be problematic. Tamminen^44^ found 14% of trigger words were focussed on consciousness. A description of a fluctuating level of consciousness decreases the chance of the OHCA being recognised and in 54% of unrecognised cases the caller gave contradictory information regarding patient consciousness.^33^

### Declarations of death

Riou et al.^56^ identified that EMDs were quicker at recognising OHCA where there was a declaration of death, but this was more likely to occur in an unwitnessed event.

### Declarations of colour change

When a patient suffers an OHCA the witness may recognise colour changes in the patient. Berdowski et al.^24^ found that in 16.5% of OHCAs the witness described a patient’s colour as blue/purple and this finding is supported by Tamminen et al.^44^ who identified that the description, ‘the patient is blue’ occurred in 18% of the true cardiac arrest group. Schwarzkoph and colleagues^42^ found that patients who have a seizure and OHCA are often described as turning blue, purple or red. Conversely Mirhaghi et al.^38^ removed ‘turning blue’ from their checklist because of a lack of frequency of occurrence, suggesting that there may be ethnic and cultural differences in the way colour change is recognised and reported during an EMS call.

### Managing the emergency call

The interaction between the caller and the EMD is vitally important and allows the EMD to triage the EMS call effectively. The EMD may not always interview the caller in the most effective way to elicit identification of OHCA.26., 30., 38., 43., 46., 49. Significant differences have been found in the way EMDs adhere to the dispatch protocol^52^ and poor adherence to the dispatch protocol has been found to be one of the main reasons why OHCA is not identified.53., 55. Research found simulation training in the management of the emergency call improved OHCA recognition and was useful for performance improvement25., 55..

Stangenes and colleagues^43^ sought to investigate whether the caller reporting a symptom versus a diagnostic condition influences EMD behaviour. The authors found that where the EMD pursued the caller’s chief complaint description before investigating breathing and consciousness there was a delay in the recognition of OHCA and the instigation of telephone CPR (tCPR). In a similar way there are significant delays to EMDs asking consciousness and breathing questions in patients who have seizure activity related to OHCA leading to delays in OHCA recognition^42^. The complete omission of questions about a patient’s breathing status was found to be a particular issue contributing to non-identification of OHCA during the EMS call.24., 30., 46., 36. In contrast, Nurmi^29^ reported that the dispatch protocol was only followed in relation to consciousness and breathing in 52% of cases, but that OHCA recognition was not higher when the protocol was adhered to. Some EMDs utilise strategies to better clarify breathing status.48., 50., 35. The Hand on Belly (HoB) technique for assessing breathing has been found to improve OHCA recognition^40^ as has the 10 s interval to assess breathing rate.^32^ Gram et al.^45^ completed a quality assessment study focussed on the introduction of a ‘No,No,Go algorithm’ (Not breathing normally, Not awake, Immediate EMS dispatch). The ‘No,No,Go algorithm’ did not improve time to asking the key questions, but the time to recognition of OHCA did improve.

Where the caller is a healthcare professional the dispatch protocol is less likely to be followed, and OHCA less likely to be recognised.^27^ Riou et al.^49^ highlight the disruption that caller pre-emption causes during the emergency call and the positive way that some EMDs employ communication techniques that help manage the pre-emption so that vital information is not lost during the call. EMDs have described the inflexibility of the dispatch protocol and a desire to ask additional questions, or to change the ordering of questions based on individual circumstance so that they can better identify OHCA.^50^

### Emotional distress

Understandably many callers who contact EMS are distressed. The studies reviewed found that in general callers are calm and cooperative during the EMS call.26., 27., 30., 46. However, relatives of the patient could only adequately describe what happened in 54% of cases compared to 72% of unrelated callers, where the caller was a doctor or nurse.^27^ Chien^26^ identified that the rate of OHCA recognition was greatest when the Emotional Content and Cooperation Score (ECCS) was the highest at 5 or 4 (ECCS 5:uncontrollable, hysterical; ECCS 4:uncooperative, not listening, yelling,^57^ suggesting that a high ECCS may indicate the presence of OHCA. These findings are supported by Hardeland et al.^52^ and Mirhaghi^38^ who report that callers convey their emotional response to the EMD indicating where the patient is in a critical condition. Conversely, the emotional response of the caller has been found to create uncertainty for EMDs46., 48., 51., 52. and make the EMS call very difficult to manage^48^ Travers and colleagues^35^ found that a calm caller can create a false reassurance and together these findings highlight the difficulties that EMDs face interpreting and navigating EMS calls.

## Discussion

This systematic mixed studies review (SMSR) set out to identify and appraise the evidence focussing on the features of the EMS call interaction that enable or inhibit an Emergency Medical Dispatcher’s recognition of a patient in out-of-hospital cardiac arrest, or at imminent risk of out-of-hospital cardiac arrest. The SMSR reviewed a broad range of evidence identifying the three main themes: Key features of the EMS call interaction, Managing the emergency call and Emotional distress.

The studies analysed demonstrate variation in practice and results across EMS systems, however a dominant finding included in the theme, “key features of the EMS call” was the importance of (and difficulty in) recognising abnormal/agonal breathing during the EMS call. Qualitative data provides context to this, describing the barriers that EMDs face in interrogating callers and recognising abnormal/agonal breathing. Qualitative data also indicates variability in practice amongst EMDs, with EMDs describing tailoring an approach to the EMS call dependent on the situation presented. It is interesting to note the focus on difficulties determining breathing status over consciousness status in the published research.

The way in which the EMD manages the EMS call is a critical factor in their ability to recognise OHCA and the deteriorating patient. Adherence to the dispatch protocol and the asking of key questions is variable with associated impacts on triage. The manner in which the caller interacts with the EMD effects the approach of the EMD to managing the EMS call and the subsequent trajectory and outcome. In addition, in some EMS systems there are strategies to clarify breathing status with varying levels of success.

The caller’s level of emotional distress impacts on the EMD and their assessment of the EMS call. The majority of callers are calm and cooperative, but high levels of emotional distress may indicate an OHCA and calm callers may create uncertainty. A highly distressed caller can make it challenging for the EMD to manage the EMS call in the most effective way.

The research question included patients who are already in OHCA at the time of the EMS call (“recognition studies”), and those patients who are not in OHCA at the time of the EMS call, but who suffer OHCA subsequently (“prediction studies”). Patients at imminent risk of cardiac arrest may be harder to identify, and it can be difficult to distinguish deteriorating and peri-arrest patients from those already in OHCA. When a patient is reported to be breathing abnormally, they could be in OHCA with agonal breathing, or they might not yet have suffered an OHCA and be breathing abnormally for other reasons. The current European Resuscitation Council Guidelines state that where there is an ‘unresponsive person with absent or abnormal breathing’ they should be assumed to be in OHCA.^58^

Unfortunately, no studies of patients at imminent risk of cardiac arrest (“prediction studies”) met the SMSR inclusion criteria. This SMSR therefore comprised studies examining EMD recognition of OHCA where the patient was known to be in cardiac arrest or their status at the time of the call was uncertain (“recognition studies”). Further research could usefully examine the features of an Emergency Medicine System call interaction that enable, or inhibit, a call taker’s recognition that a patient who is unequivocally alive during the EMS call is at imminent risk of OHCA. The effective identification of a person at imminent risk of OHCA will allow EMS to respond in an optimum way with the aim of improving survival in this important patient group.

Meta-analysis of quantitative findings and meta-synthesis of qualitative findings in systematic reviews consists of well-established methods for combining results and data across studies.^16^ Completing systematic reviews where the results of qualitative, quantitative and mixed methods studies are presented in a single systematic review is relatively new and presents the challenge of data integration between these diverse study types.^16^ In SMSRs there is methodological diversity, within and between studies.^15^

A strength of this SMSR is the diverse range of papers included. Papers were included from a range of different regions, cultures and EMS systems. International EMS systems are adapted to local societal, cultural and financial factors^53^ and some findings may not be generalisable to alternative cultures and EMS settings. The quantitative papers identified did not lend themselves to meta-analysis due to heterogeneity of studies. Similarly, qualitative papers did not lend themselves to meta-synthesis. The many different types of studies included in this SMSR reflect the wide range of approaches researchers have taken to generate knowledge in this area. Although challenging, it is important to synthesise all available knowledge so that fully evidence-based recommendations can be made.

Due to the heterogeneity of the studies included, the most recent version of the MMAT^23^ was used to critically appraise the included papers. The reliability of the previous MMAT (2011 version)^59^ has been appraised by Souto and colleagues and Pace and colleagues.18., 60. The appraisal confirmed the MMAT as an efficient tool, but with improvements required in its reliability. Discrepancies were found in reviewers’ interpretations of aspects of the tool. Also, some qualitative research papers had limited mention of some items, including the documentation of reflexivity and how findings relate in the context. In this SMSR there was no disagreement between reviewers regarding quality assessment. The MMAT 2018 has been revised to reflect appraisal of the MMAT 2011, but the authors acknowledge the requirement for further testing of reliability and validity in the future.^12^

A quantifiable scale was chosen to score the included papers using the MMAT. However this is discouraged in the MMAT manual, with a preference for reviewers to provide more details of the ratings for each paper.^23^ Other SMSR reviewers have set a precedent of scoring using the MMAT in the way that was followed in this review.19., 20., 21., 22. The decision to use quantitative scoring was compensated for by providing detail in the limitations section for each paper recorded in the results, supplementary Tables S3–9.

A limitation to consider is that this SMSR was limited to English language studies. The PRISMA study flow diagram in Appendix Two indicates two papers were excluded because they were non-English, and this data has been lost to this review.

## Recommendations for further research

Further research that investigates the EMS call interaction of those patients who are not in OHCA at the time of the call and then deteriorate into OHCA subsequently is required to better understand the features of this patient group, and improve dispatch. Larger studies are recommended that investigate which communication strategies and interventions in which context allow the EMD to interrogate the caller most effectively. EMD training is important, and further research is required to investigate which methods of training are most appropriate to enable EMDs to manage the challenges of triage in this high-risk patient group. This review highlights the relative absence of research focusing on consciousness in OHCA compared to abnormal breathing, with a need for more research in this area.

## Conclusions

The first link in the chain of survival; early recognition of OHCA and call for help, is a critical first stage as it enables a sequence of events to be put into action that can ultimately save a person’s life. This SMSR reviewed 32 primary research studies. A main finding was the importance of recognising abnormal/agonal breathing and the difficulties that EMDs face in recognising abnormal/agonal breathing during the EMS call.

This SMSR highlights an absence of research examining the EMS call interaction with patients who are not in OHCA when the EMS call is made, but who deteriorate into OHCA subsequently. Recommendations for future research focus on EMD communication strategies, EMD training and the development of interventions that allow EMDs to better predict which patients will deteriorate into OHCA following an EMS call.

## Role of the funding source

The lead author, Kim Kirby, is completing a Clinical Doctoral Research Fellowship funded by the National Institute for Health Research in the UK (ICA-CDRF-2018-04-ST2-007). This manuscript is an output from this fellowship funding.

## Declaration of conflicts of interest

Conflicts of interest: none.

## Source of Support

The lead author is completing a Clinical Doctoral Research Fellowship funded by the National Institute for Health Research in the UK.

## Figures and Tables

**Table:** 

Medline Search Strategy May 2021		
1	“HEART ARREST”/ OR “OUT-OF-HOSPITAL CARDIAC ARREST”/	34,081
2	(out-of-hospital cardiac arrest).ti,ab	6,982
3	(out of hospital cardiac arrest).ti,ab	7,467
4	(heart arrest).ti,ab	10,721
5	(out-of-hospital heart arrest).ti,ab	779
6	(out of hospital heart arrest).ti,ab	905
7	(cardiac arrest).ti,ab	39,902
8	(OHCA).ti,ab	2,913
9	(OOHCA).ti,ab	76
10	*(1 OR 2 OR 3 OR 4 OR 5 OR 6 OR 7 OR 8 OR 9)*	57,295
11	(“EMS call”).ti,ab	72
12	(“Emergency Medical Service call”).ti,ab	12
13	(“999 call”).ti,ab	29
14	(“112 call”).ti,ab	7
15	(“911 call”).ti,ab	72
16	(“emergency call”).ti,ab	469
17	(“emergency medical system call”).ti,ab	2
18	(“emergency medical call”).ti,ab	11
19	(dispatch*).ti,ab	3504
20	*(11 OR 12 OR 13 OR 14 OR 15 OR 16 OR 17 OR 18 OR 19)*	3991
21	*(10 AND 20)*	810

**Figure:**
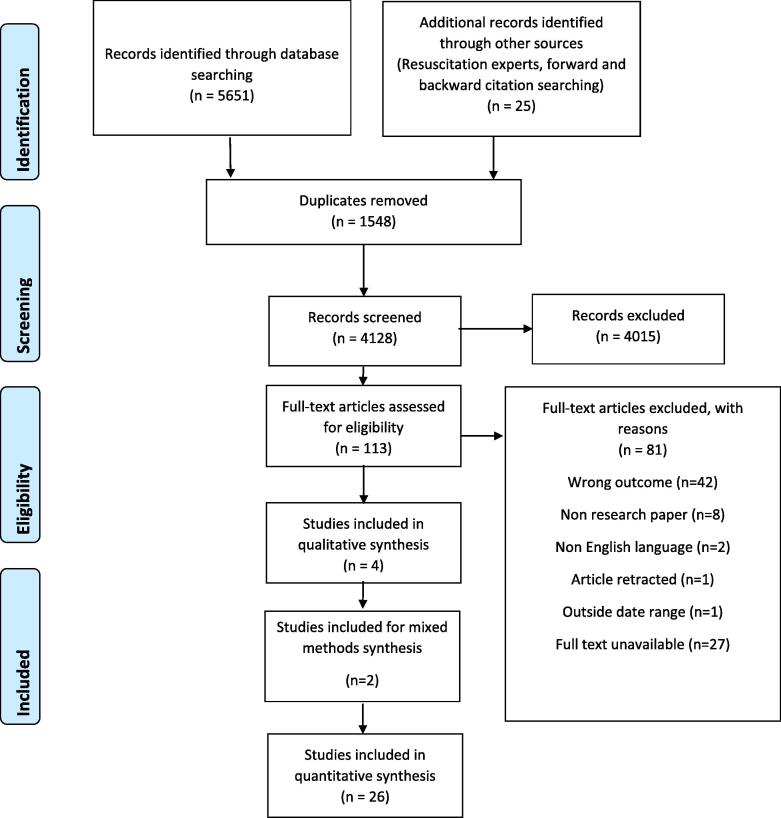


## References

[1] Yan S., Gan Y., Jiang N. (2020). The global survival rate among adult out-of-hospital cardiac arrest patients who received cardiopulmonary resuscitation: a systematic review and meta-analysis. Crit Care.

[2] Deakin CD, Brown S, Jewkes F, et al. Guidelines: Prehospital resuscitation. Resuscitation Council UK; Published 2015. https://www.resus.org.uk/library/2015-resuscitation-guidelines/prehospital-resuscitation [accessed August 18, 2020].

[3] Nolan J., Soar J., Eikeland H. (2006). The chain of survival. Resuscitation.

[4] Lancet T. (2018). Out-of-hospital cardiac arrest: a unique medical emergency. Lancet.

[5] NHS England, British Heart Foundation, Resuscitation Council (UK). Consensus Paper on Out-of-Hospital Cardiac Arrest in England; Published 2014. https://www.resus.org.uk/sites/default/files/2020-05/OHCA_consensus_paper.pdf [accessed May 21, 2021].

[6] American Heart Association. Telecommunicator CPR (T-CPR). cpr.heart.org; Published 2019. https://cpr.heart.org/en/resuscitation-science/telecommunicator-cpr [accessed August 18, 2020].

[7] Deakin C.D. (2018). The chain of survival: Not all links are equal. Resuscitation.

[8] Perkins G.D., Lockey A.S., de Belder M.A., Moore F., Weissberg P., Gray H. (2016). National initiatives to improve outcomes from out-of-hospital cardiac arrest in England. Emerg Med J.

[9] Kleinman M.E., Perkins G.D., Bhanji F. (2018). ILCOR Scientific Knowledge Gaps and Clinical Research Priorities for Cardiopulmonary Resuscitation and Emergency Cardiovascular Care: A Consensus Statement. Circulation.

[10] Drennan I.R., Geri G., Brooks S. (2021). Diagnosis of out-of-hospital cardiac arrest by emergency medical dispatch: A diagnostic systematic review. Resuscitation.

[11] Vaillancourt C., Charette M.L., Bohm K., Dunford J., Castrén M. (2011). In out-of-hospital cardiac arrest patients, does the description of any specific symptoms to the emergency medical dispatcher improve the accuracy of the diagnosis of cardiac arrest: A systematic review of the literature. Resuscitation.

[12] Hong Q.N., Fàbregues S., Bartlett G. (2018). The Mixed Methods Appraisal Tool (MMAT) version 2018 for information professionals and researchers. Educ Inf.

[13] Pluye P., Gagnon M.-P., Griffiths F., Johnson-Lafleur J. (2009). A scoring system for appraising mixed methods research, and concomitantly appraising qualitative, quantitative and mixed methods primary studies in Mixed Studies Reviews. Int J Nurs Stud.

[14] Pluye P., Hong Q.N. (2014). Combining the Power of Stories and the Power of Numbers: Mixed Methods Research and Mixed Studies Reviews. Annu Rev Public Health.

[15] Sandelowski M., Voils C.I., Barroso J. (2006). Defining and Designing Mixed Research Synthesis Studies. Res Sch Natl Refereed J Spons -South Educ Res Assoc Univ Ala.

[16] Frantzen K.K., Fetters M.D. (2016). Meta-integration for synthesizing data in a systematic mixed studies review: insights from research on autism spectrum disorder. Qual Quant.

[17] Sandelowski M., Voils C.I., Leeman J., Crandell J.L. (2012). Mapping the Mixed Methods-Mixed Research Synthesis Terrain. J Mix Methods Res.

[18] Pace R., Pluye P., Bartlett G. (2012). Testing the reliability and efficiency of the pilot Mixed Methods Appraisal Tool (MMAT) for systematic mixed studies review. Int J Nurs Stud.

[19] Massey D., Chaboyer W., Anderson V. (2017). What factors influence ward nurses’ recognition of and response to patient deterioration? An integrative review of the literature. Nurs Open.

[20] Clifford B.K., Mizrahi D., Sandler C.X. (2018). Barriers and facilitators of exercise experienced by cancer survivors: a mixed methods systematic review. Support Care Cancer.

[21] Mey A., Plummer D., Dukie S., Rogers G.D., O’Sullivan M., Domberelli A. (2017). Motivations and Barriers to Treatment Uptake and Adherence Among People Living with HIV in Australia: A Mixed-Methods Systematic Review. AIDS Behav.

[22] Scott S.D., Rotter T., Flynn R. (2019). Systematic review of the use of process evaluations in knowledge translation research. Syst Rev.

[23] Hong QN, Pluye P, Fabregues S, et al. MMAT_2018_criteria-manual_2018-08-01_ENG.pdf; Published 2018. http://mixedmethodsappraisaltoolpublic.pbworks.com/w/file/fetch/127916259/MMAT_2018_criteria-manual_2018-08-01_ENG.pdf [accessed September 17, 2020].

[24] Berdowski J., Beekhuis F., Zwinderman A.H., Tijssen J.G.P., Koster R.W. (2009). Importance of the first link: description and recognition of an out-of-hospital cardiac arrest in an emergency call. Circulation.

[25] Meischke H., Painter I.S., Stangenes S.R. (2017). Simulation training to improve 9–1-1 dispatcher identification of cardiac arrest: A randomized controlled trial. Resuscitation.

[26] Chien C.-Y., Chien W.-C., Tsai L.-H. (2019). Impact of the caller’s emotional state and cooperation on out-of-hospital cardiac arrest recognition and dispatcher-assisted cardiopulmonary resuscitation. Emerg Med J EMJ.

[27] Castrén M., Kuisma M., Serlachius J., Skrifvars M. (2001). Do health care professionals report sudden cardiac arrest better than laymen?. Resuscitation.

[28] Garza A.G., Gratton M.C., McElroy J., Lindholm D., Coontz D. (2008). Environmental factors encountered during out-of-hospital intubation attempts. Prehosp Emerg Care.

[29] Nurmi J., Pettilä V., Biber B., Kuisma M., Komulainen R., Castrén M. (2006). Effect of protocol compliance to cardiac arrest identification by emergency medical dispatchers. Resuscitation.

[30] Ma M.H.-M., Lu T.-C., Ng J.C.-S. (2007). Evaluation of emergency medical dispatch in out-of-hospital cardiac arrest in Taipei. Resuscitation.

[31] Clawson J., Olola C., Scott G., Heward A., Patterson B. (2008). Effect of a Medical Priority Dispatch System key question addition in the seizure/convulsion/fitting protocol to improve recognition of ineffective (agonal) breathing. Resuscitation.

[32] Roppolo L.P., Westfall A., Pepe P.E. (2009). Dispatcher assessments for agonal breathing improve detection of cardiac arrest. Resuscitation.

[33] Lewis M., Stubbs B.A., Eisenberg M.S. (2013). Dispatcher-assisted cardiopulmonary resuscitation: time to identify cardiac arrest and deliver chest compression instructions. Circulation.

[34] Hardeland C., Olasveengen T.M., Lawrence R. (2014). Comparison of Medical Priority Dispatch (MPD) and Criteria Based Dispatch (CBD) relating to cardiac arrest calls. Resuscitation.

[35] Travers S., Jost D., Gillard Y. (2014). Out-of-hospital cardiac arrest phone detection: those who most need chest compressions are the most difficult to recognize. Resuscitation.

[36] Møller T.P., Andréll C., Viereck S., Todorova L., Friberg H., Lippert F.K. (2016). Recognition of out-of-hospital cardiac arrest by medical dispatchers in emergency medical dispatch centres in two countries. Resuscitation.

[37] Biancardi M.A.A., Spiteri P., Pace M.P. (2017). Cardiac arrest recognition and telephone CPR by emergency medical dispatchers. Malta Med Sch Gaz.

[38] Mirhaghi A., Shafaee H., Malekzadeh J., Hasanzadeh F. (2017). Recognizing Sudden Cardiac Arrest May Require More Than Two Questions during Telephone Triage: Developing a Complementary Checklist. Bull Emerg Trauma.

[39] Riou M., Ball S., Williams T.A. (2018). ‘She’s sort of breathing’: What linguistic factors determine call-taker recognition of agonal breathing in emergency calls for cardiac arrest?. Resuscitation.

[40] Derkenne C., Jost D., Thabouillot O. (2020). Improving emergency call detection of Out-of-Hospital Cardiac Arrests in the Greater Paris area: Efficiency of a global system with a new method of detection. Resuscitation.

[41] Mao D.R., Ee A.Z.Q., Leong P.W.K. (2020). Is your unconscious patient in cardiac arrest? A New protocol for telephonic diagnosis by emergency medical call-takers: A national study. Resuscitation.

[42] Schwarzkoph M., Counts C.R., Eisenberg M., Yin L., Hergert L., Drucker C. (2020). Seizure-like presentation in OHCA creates barriers to dispatch recognition of cardiac arrest. Resuscitation.

[43] Stangenes S.R., Painter I.S., Rea T.D., Meischke H. (2020). Delays in recognition of the need for telephone-assisted CPR due to caller descriptions of chief complaint. Resuscitation.

[44] Tamminen J., Lydén E., Kurki J., Huhtala H., Kämäräinen A., Hoppu S. (2020). Spontaneous trigger words associated with confirmed out-of-hospital cardiac arrest: a descriptive pilot study of emergency calls. Scand J Trauma Resusc Emerg Med.

[45] Gram K.H., Præst M., Laulund O., Mikkelsen S. (2021). Assessment of a quality improvement programme to improve telephone dispatchers’ accuracy in identifying out-of-hospital cardiac arrest. Resusc Plus.

[46] Bång A., Herlitz J., Martinell S. (2003). Interaction between emergency medical dispatcher and caller in suspected out-of-hospital cardiac arrest calls with focus on agonal breathing. A review of 100 tape recordings of true cardiac arrest cases. Resuscitation.

[47] Bohm K., Stålhandske B., Rosenqvist M., Ulfvarson J., Hollenberg J., Svensson L. (2009). Tuition of emergency medical dispatchers in the recognition of agonal respiration increases the use of telephone assisted CPR. Resuscitation.

[48] Bång A., Ortgren P.-O., Herlitz J., Währborg P. (2002). Dispatcher-assisted telephone CPR: a qualitative study exploring how dispatchers perceive their experiences. Resuscitation.

[49] Riou M., Ball S., O’Halloran K.L., Whiteside A., Williams T.A., Finn J. (2018). Hijacking the dispatch protocol: When callers pre-empt their reason-for-the-call in emergency calls about cardiac arrest. Discourse Stud.

[50] Jensen J.L., Vaillancourt C., Tweedle J. (2012). Factors Associated with the Successful Recognition of Abnormal Breathing and Cardiac Arrest by Ambulance Communications Officers: A Qualitative Iterative Survey. Prehosp Emerg Care.

[51] Alfsen D., Møller T.P., Egerod I., Lippert F.K. (2015). Barriers to recognition of out-of-hospital cardiac arrest during emergency medical calls: a qualitative inductive thematic analysis. Scand J Trauma Resusc Emerg Med.

[52] Hardeland C., Sunde K., Ramsdal H. (2016). Factors impacting upon timely and adequate allocation of prehospital medical assistance and resources to cardiac arrest patients. Resuscitation.

[53] Watkins C.L., Jones S.P., Hurley M.A. (2021). Predictors of recognition of out of hospital cardiac arrest by emergency medical services call handlers in England: a mixed methods diagnostic accuracy study. Scand J Trauma Resusc Emerg Med.

[54] Garza A.G., Gratton M.C., Chen J.J., Carlson B. (2003). The accuracy of predicting cardiac arrest by emergency medical services dispatchers: the calling party effect. Acad Emerg Med.

[55] Hardeland C., Skåre C., Kramer-Johansen J. (2017). Targeted simulation and education to improve cardiac arrest recognition and telephone assisted CPR in an emergency medical communication centre. Resuscitation.

[56] Riou M., Ball S., Morgan A. (2021). “I think he’s dead”: A cohort study of the impact of caller declarations of death during the emergency call on bystander CPR. Resuscitation.

[57] Clawson J.J., Sinclair R. (2001). The emotional content and cooperation score in emergency medical dispatching. Prehospital Emerg Care Off J Natl Assoc EMS Physicians Natl Assoc State EMS Dir.

[58] Olasveengen T.M., Mancini M.E., Perkins G.D. (2020). Adult Basic Life Support: International Consensus on Cardiopulmonary Resuscitation and Emergency Cardiovascular Care Science With Treatment Recommendations. Resuscitation.

[59] Pluye P, Robert E, Cargo M, et al. MMAT_2011_criteria_and_tutorial_2011-06-29updated2014.08.21.pdf. Published 2011. http://mixedmethodsappraisaltoolpublic.pbworks.com/w/file/fetch/84371689/MMAT_2011_criteria_and_tutorial_2011-06-29updated2014.08.21.pdf [accessed January 7, 2021].

[60] Souto R.Q., Khanassov V., Hong Q.N., Bush P.L., Vedel I., Pluye P. (2015). Systematic mixed studies reviews: updating results on the reliability and efficiency of the Mixed Methods Appraisal Tool. Int J Nurs Stud.

