# Supplementary material for: Features of Emergency Medical System calls that facilitate or inhibit Emergency Medical Dispatcher recognition that a patient is in, or at imminent risk of, cardiac arrest: A systematic mixed studies review
Source: Resusc Plus. 2021 Nov 18;8:100173. doi: 10.1016/j.resplu.2021.100173 (PMC8605417; doi:10.1016/j.resplu.2021.100173)
Supplement: Supplementary data 1 [file mmc1.docx]

Supplementary Table S1

Categories of study design

| **Category of Study Design** | **Number of Papers** | **References** |
| --- | --- | --- |
| Qualitative | 4 | (Alfsen et al., 2015^51^, Bång et al., 2002^48^; Jensen et al., 2012^50^; Riou et al., 2018^49^) |
| Quantitative Randomised Controlled Trial | 1 | (Meischke et al., 2017^25^) |
| Quantitative Non-Randomised Controlled Trial | 11 | (Chien et al., 2019^26^; Clawson et al., 2008^31^; Derkenne et al., 2020^40^; Gram et al., 2021^45^; Hardeland et al., 2014^34^, 2017^55^; Lewis et al., 2013^33^; Mao et al., 2020^41^; Riou et al., 2021^56^; Roppolo et al., 2009^32^; Schwarzkoph et al., 2020^42^) |
| Quantitative Descriptive | 14 | (Bång et al., 2003^46^; Berdowski et al., 2009^24^; Biancardi et al., 2017^37^; Bohm et al., 2009^47^; Castrén et al., 2001^27^; Garza et al., 2003^28^; Ma et al., 2007^30^; Mirhaghi et al., 2017^38^; Møller et al., 2016^36^; Nurmi et al., 2006^29^; Riou et al., 2018^39^; Stangenes et al., 2020^43^; Tamminen et al., 2020^44^; Travers et al., 2014^35^) |
| Mixed Methods | 2 | (Hardeland et al., 2016^52^; Watkins C.L. et al., 2021^53^) |

Supplementary Table S2

Grading of papers

|  | High Certainty | Moderate Certainty | Low Certainty |
| --- | --- | --- | --- |
| Quantitative | 3 papers. (Berdowski et al., 2009^24^; Chien et al., 2019^26^; Meischke et al., 2017^25^) | 21 papers (Biancardi et al., 2017^37^; Castrén et al., 2001^27^; Clawson et al., 2008^31^; Derkenne et al., 2020^40^; Garza et al., 2003^28^; Gram et al.,2021^45^; Hardeland et al., 2014^34^, 2017^55^; Lewis et al., 2013^33;^ Ma et al., 2007^30^; Mao et al., 2020^41^ Mirhaghi et al., 2017^38^; Møller et al., 2016^36^; Nurmi et al., 2006^29^; Riou, et al., 2018^39^,2021^56^; Roppolo et al., 2009^32^; Schwarzkoph et al., 2020^42;^ Stangenes et al., 2020^43^; Tamminen et al., 2020^44^; Travers et al., 2014^35^) | 2 papers (Bång et al., 2003^46^; Bohm et al., 2009^47^) |
| Qualitative | 2 papers (Bång et al., 2002^48^; Riou et al., 2018^49^) | 2 papers (Alfsen et al., 2015^51^; Jensen et al., 2012^50^) |  |
| Mixed Methods | 1 paper (Hardeland et al., 2016^52^) | 1 paper (Watkins et al.2021^53^ |  |

Supplementary Table S3

*Quantitative Papers (Grouped by certainty) and listed in descending order of date of publication*

**High Certainty Papers**

| **Quantitative Descriptive Study** |  | | | | | |
| --- | --- | --- | --- | --- | --- | --- |
| **Berdowski (2009) Importance of the first link: Description and recognition of OHCA in an emergency call^24^** | | | | | | |
| **Study Design**  **Aim** | **Date of Data Collection** | **Setting/**  **Participants** | **Main Findings** | **Dispatch Software** | **Limitations** | **Quality Grade**  MMAT 2018 |
| Prospective Observational Study.  To investigate the recognition of OHCA on the emergency call. | Jan 2004 – Sep 2004 | Amsterdam, Netherlands.  Consecutive high priority emergency calls to the Greater Amsterdam Dispatch Unit, where a layperson was the caller. | OHCA recognition sensitivity 71%.  Specificity 99.3%.  The 82 patients with a cardiac arrest not recognised by the dispatcher showed lower survival rates compared with the 203 patients with recognised cardiac arrests, as shown in an unadjusted Kaplan-Meier model (log-rank P 0.04).  When a cardiac arrest was suspected, the mean time interval between call and dispatching was 1.88 minutes (SD,1.10 minutes) versus 2.82 minutes (SD, 1.60 minutes) when cardiac arrest was not suspected (P0.001).  Mean time interval from call to arrival also differed significantly: 8.55 minutes (SD, 4.93 minutes) for calls with cardiac arrest suspicion versus 9.95 minutes (SD, 3.73 minutes) for calls without cardiac arrest suspicion (P0.01).  When not recognising the cardiac arrest, the dispatcher did not ask about the patient’s breathing in 42 calls (51%); the caller gave a positive answer when asked about presence of breathing in 16 calls (20%); and the patient was reported to breathe abnormally in 20 calls (24%). For the calls in which cardiac arrests were recognised, these numbers were 51(25%), 10 (5%), and 41(20%), respectively. Asking for breathing differed significantly in these groups (P=0.001).  Description of the facial colour as blue or purple occurred in 16.5% of OHCAs and contributed substantially to the probability of a cardiac arrest (p=0.001).  Abnormal breathing was described in 40% of the calls in which the dispatcher asked if the patient was breathing. In those patients, prevalence of cardiac arrest was 32%. | Unclear | Unclear which dispatch system was used for triaging calls and whether the results are generalisable to other systems. | High |
| **Randomised Controlled Trial** |  | | | | | |
| **Meischke (2017) Simulation training to improve 9-1-1 dispatcher identification of cardiac arrest: A randomised controlled trial^25^** | | | | | | |
| **Study Design**  **Aim** | **Date of Data Collection** | **Setting/**  **Participants** | **Main Findings** | **Dispatch Software** | **Limitations** | **Quality Grade**  MMAT 2018 |
| A parallel prospective randomised controlled trial.  To determine if simulation training improved OHCA identification and time to T-CPR for EMDs using a CBD guideline-based dispatch system. | Sep 2013 – Apr 2016 | US.  128 EMDs from 13  9-1-1 call centres in the states of Washington, Oregon, Alaska and Arizona. | In seven of the 256 assessment calls, the participant did not recognise the need for T-CPR; Recognition of the need for T-CPR was greater in the intervention compared to the control group (100% versus 94%, p = 0.01).  The mean time to transition to T-CPR was 21 s less in the intervention group than the control group (60 vs. 81 s, p < 0.001). Similarly, time to instruction was 23 s less in the intervention group than the control group (73 vs 91 s, p < 0.001). These times were similar for each of the two scripts used in the assessment.  When assessing performance on real life OHCA calls, after adjusting for call centre, comparing the performance of EMDs who had completed the study protocol of 4 simulation trainings with EMDs who had less than 4 trainings showed statistically significant differences for label (p = 0.022) and consciousness (p = 0.041), indicating that receiving 4 trainings was associated with better performance on these skills. | Criteria-Based Dispatch | The study findings may not be generalisable to non-CBD dispatch systems.  May not be generalisable to other EMD centres.  Confounders not considered.  The length of effect of intervention not taken into consideration. | High |
| **Quantitative non-randomised study** |  | | | | | |
| **Chien (2019) Impact of the caller’s emotional state and co-operation on out-of-hospital cardiac arrest recognition and dispatcher-assisted cardiopulmonary resuscitation^26^** | | | | | | |
| **Study Design**  **Aim** | **Date of Data Collection** | **Setting/**  **Participants** | **Main Findings** | **Dispatch Software** | **Limitations** | **Quality Grade**  MMAT 2018 |
| Retrospective Cross-Sectional Study.  To examine the association between callers’ Emotional Content and Cooperation Score (ECCS) and dispatchers’ OHCA recognition and DA-CPR instruction performance. | Nov 2015-Oct 2016 | Northern Taiwan  Emergency call audio recordings of documented adult, non-traumatic OHCA.  2015/2016 | Dispatchers recognised OHCA in 251 (68.4%) cases.  Unambiguous responses about the patient’s consciousness and breathing status were received in 343 (93.5%) and 281 (76.6%) cases, respectively.  Only 8.4% of the callers rated as ECCS 4–5.  The rate of OHCA recognition was the greatest in the ECCS 4–5 group. These results suggest that a high ECCS level can be a preliminary clue for the dispatcher for recognising OHCA.  An unambiguous response for patient consciousness was similar across the ECCS levels (92.7%–95.7%), whereas the unambiguous response rate for patient breathing status was highest in the ECCS 3 (84.8%), followed by ECCS 2 (84.0%), ECCS 4–5 (77.4%) and ECCS 1 (68.5%) caller groups.  Dispatchers failed to recognise OHCA in 30 cases where an unambiguous response to the patient’s breathing status was obtained.  The rate of OHCA recognition by dispatchers increased with ECCS level: ECCS 1 (61.8%), ECCS 2 (72.0%), ECCS 3 (76.1%), ECCS 4–5 (77.4%).  Dispatcher error contributed mostly to the reason for non-recognition of OHCA in the low ECCS groups (ECCS 1: 73%, ECCS 2: 77%).  In multivariable analysis, dispatchers were significantly more likely to receive unambiguous responses about the patient’s breathing status from callers classified as ECCS 2 (adjusted OR (AOR)=2.6, 95%CI 1.4 to 4.7) and ECCS 3 (AOR=2.6, 95%CI 1.1 to 6.4).  Dispatcher OHCA recognition was significantly associated with the ECCS 3 group (AOR=2.3,95%CI 1.1 to 5.0).  The cooperative caller group had a significantly higher successful delivery rate of DA-CPR instruction than the uncooperative caller group (85.9%vs 54.2%, p<0.01).  The median times to OHCA recognition, CPR instruction and chest compression were 38.0, 80.5 and 170.0s, respectively, in the cooperative caller group and 29.0, 91.5 and 122.0s, respectively, in the uncooperative caller group. | Unclear | May not be generalisable outside of Mandarin speaking population.  Small sample size.  Possible bias in assessing ECCS. | High |

Supplementary Table S4

*Quantitative Papers (Grouped by certainty) and listed in descending order of date of publication*

**Moderate Certainty Quantitative Papers**

| **Quantitative Descriptive Study** |  | | | | | | |
| --- | --- | --- | --- | --- | --- | --- | --- |
| **Castren (2001) Do health care professionals report sudden cardiac arrest better than laymen?^27^** | | | | | | | |
| **Study Design**  **Aim** | **Date of Data Collection** | | **Setting/**  **Participants** | **Main Findings** | **Dispatch Software** | **Limitations** | **Quality Grade**  MMAT 2018 |
| Prospective study.  To compare the emergency calls made by health care providers and by laymen reporting a non-traumatic cardiac arrest, and to evaluate the handling of these calls by dispatchers. | Jan 1996 – Dec 1996 | | Helsinki, Finland.  Helsinki EMDs. | **Group 1 Doctors and Nurses**  70% cardiac arrests recognised.  Survival to hospital discharge 21%.  The dispatcher asked less questions and more likely to abandon protocol and only asked 10% of these callers to do CPR.  **Group 2 Other Health Care professionals**  74% cardiac arrests recognised.  Survival to hospital discharge 32%.  **Group 3 Laymen**  73% of cardiac arrests recognised.  Survival to hospital discharge 28%.  21% asked by dispatchers to do CPR.  Callers were usually calm and cooperative.  TCPR instructions were given to 27% of the laymen and to 40% of relatives calling, but to only 2% of the professional callers. | Criteria-Based Dispatch | 276 in group 3 compared to just 33 in group 1. | Moderate |
| **Quantitative Descriptive Study** |  | | | | | | |
| **Garza (2003) The accuracy of predicting cardiac arrest by emergency medical services dispatchers: the calling party effect^28^** | | | | | | | |
| **Study Design**  **Aim** | **Date of Data Collection** | | **Setting/**  **Participants** | **Main Findings** | **Dispatch Software** | **Limitations** | **Quality Grade**  MMAT 2018 |
| Retrospective Review of EMS Dispatch Data.  To analyse the accuracy of EMS dispatchers in predicting cardiac arrest and to assess the effect of the caller party on dispatcher accuracy in an advanced life support. | Jan 2000 – Jun 2000 | | US.  Midwest urban EMS system using AMPDS. | The sensitivity for a code of OHCA given was 68.3% (95% CI 3.3% to 73.0%) with a PPV of 65.0% (95% CI 60.0%to 69.7%). Fourth-party callers had the highest sensitivity at 74.29% (95% CI 62.2% to 83.7%), whereas second-party callers had the highest PPV at 71.67% (95% CI 65.3% to 77.3%). Chi-square analysis comparing the sensitivity between the calling parties was not signiﬁcant (x^2^ = 3.728, 2 df, p=0.17).  The appropriateness of the ﬁnal coding was scored at 94.78% (95% CI 92.63% to 97.79%), meaning the quality improvement reviewer agreed that the correct dispatch code was given approximately 95% of the time. The overall dispatch protocol compliance score was 85.22 % (95% CI 83.33% to 87.10%), meaning the dispatcher followed the protocol according to the EMD standards, about 85% of the time. There was no signiﬁcant statistical difference in scores between the calling parties for ﬁnal coding (p =0.88) or in protocol compliance (p =0.37). | Advanced Medical Priority Dispatch System | Retrospective data collection.  Unable to check accuracy of paramedic field diagnosis of OHCA. | Moderate |
| **Quantitative Descriptive Study** |  | | | | | | |
| **Nurmi (2006) Effect of protocol compliance to cardiac arrest identification by emergency medical dispatchers^29^** | | | | | | | |
| **Study Design**  **Aim** | **Date of Data Collection** | | **Setting/**  **Participants** | **Main Findings** | **Dispatch Software** | **Limitations** | **Quality Grade**  MMAT 2018 |
| Prospective Study.  The objective of the study was to assess the effect of protocol compliance to the accuracy of cardiac arrest (CA) identification by the dispatchers. | 1996. | | Helsinki, Finland. | The numbers of correctly identified true witnessed (Group 1) and unwitnessed (Group 2) CAs were 122 and 441, respectively. One hundred and sixteen patients were not identified as CA by the dispatchers (17%) but were found to be lifeless when the ambulance crew arrived at the patient (false negative, Group 3), including CAs missed by the dispatcher as well as patients arrested while waiting for the EMS unit to arrive. Ninety-seven calls were incorrectly categorised as CA by the dispatcher and the patient was not in CA when the ambulance crew arrived (false positive, Group 4). The patients falsely categorized as CA were commonly patients with transient ischaemic attack, stroke, intoxication, syncope or seizures.  Information about consciousness and breathing, required by the protocol, was gathered in 52.4% of all emergency calls, more often in witnessed than in unwitnessed cases (72.3% versus 45.0%, P < 0.001).  The cardiac arrest identification rate was not significantly higher when the protocol was adhered to in witnessed cases (80.4% versus 74.4%, P = 0.5111).  In unwitnessed cases, the identification rate was lower when the protocol was adhered to (79.7% versus 87.8%, P = 0.0117) cases.  In the group of unidentified cardiac arrests (Group 3) the protocol was adhered to in 60.3%, more often in witnessed (66.7%) than in unwitnessed (57.8%) cases.  In witnessed cases where the protocol was adhered to, the delay to dispatching a first unit was shorter (median, 71 s versus 91 s, P < 0.0001), but there was no significant difference in dispatching delay of MICU (median, 157 s versus 132 s, P = 0.4039).  The Bayesian analysis revealed that from all 42 points gathered from every call only three question/answer combinations were associated independently with CA, namely: (1) consciousness; (2) breathing normally; (3) having seizures.  The identification rate of CA was 69% when breathing was not described and rose to 80% when described as abnormal and to 89% when described as absent. | Criteria-Based Dispatch | Possible errors and bias introduced when extracting audio data.  Data included patients arresting between the start of the emergency call and EMS arrival on scene.  Data collected in 1996.  Criteria based dispatch system. Results may not be generalisable to other dispatch systems. | Moderate |
| **Quantitative Descriptive Study** |  | | | | | | |
| **Ma (2007) Evaluation of emergency medical dispatch in out-of-hospital cardiac arrest in Taipei^30^** | | | | | | | |
| **Study Design**  **Aim** | **Date of Data Collection** | | **Setting/**  **Participants** | **Main Findings** | **Dispatch Software** | **Limitations** | **Quality Grade**  MMAT 2018 |
| Retrospective Observational Study.  Review of dispatch audio recordings to examine the emotional content and cooperation score (ECCS)  among Mandarin Chinese speaking callers for cardiac arrests, and evaluate the  performance of the EMS dispatching system. | Jan 2004-Apr 2004 | | Tapei.  Taipei Fire Department Dispatching Centre.  Uses a simplified version of the priority dispatch tool. | The sensitivity and positive predictive value (PPV) for predicting OHCA by dispatchers was 96.9% and 97.9%, respectively.  The average ECCS was low at 1.42 in this Mandarin speaking population.  The level of consciousness was not asked about it in 75 cases (55% = 75/137).  The breathing status was not raised in 56 cases (32% = 56/175). Of the 119 cases, actual breathing status was determined in 91 cases and in 28 cases it was unclear.  Only 38% of OHCA patient received on scene CPR, including bystander initiated (n = 13, 6%) and T-CPR provided by dispatchers (n = 17, 9%) or duty nurse in the dispatching centre (n = 45, 23%). Among those calls without CPR (n = 124, 62%), T-CPR was not offered by the dispatchers in 113 cases (57%) and 11 callers (5%) were not willing to perform T-CPR.  Of the cases analysed, 79 interviews (39.7%) were optimal (5 points) and 85 (42.7%) close to optimal (4 points) regarding the dispatcher’s interview skills. In 32 (17.6%) cases, the interview skill was suboptimal (3 points). Three cases (1.5%) were deemed to be unacceptable (2 points). | Simplified Priority Dispatch Protocol | Small sample.  Unclear if ‘targeting’ of cases may have introduced selection bias.  Unclear how data was linked from ‘field diagnosis’ to obtain call data.  May have been bias through missing data. 50/30 calls excluded due to missing data.  Interview checklist is an unvalidated tool.  Questionable whether the results are generalisable outside of the Mandarin speaking population. | Moderate |
| **Quantitative non-randomised study** |  | | | | | | |
| **Clawson (2008)** **Effect of a Medical Priority Dispatch System key question addition in the seizure/convulsion/fitting protocol to improve recognition of ineffective (agonal) breathing^31^** | | | | | | | |
| **Study Design**  **Aim** | **Date of Data Collection** | | **Setting/**  **Participants** | **Main Findings** | **Dispatch Software** | **Limitations** | **Quality Grade**  MMAT 2018 |
| Retrospective Comparative Study - Before and after study comparing a changed MPDS protocol with updated breathing question.  To investigate the impact of a new assessment question in the MPDS seizure protocol on the ability of the EMDs to identify the presence of agonal or ineffective breathing. | 2004-2006 | | London  OHCA patients and those 'Blued In' (paramedic declaration of high acuity) | Within v11.2 the odds of OHCA outcome in the 12-A-1 code was significantly reduced by 50% (OR (95%CI):0.5(0.29,0.85), p=0.009.  OHCA outcome in v11.2 was almost twice more likely in combined delta codes than in all other protocol 12 descriptor codes (OR (95%):2.10(1.30,1.40), p=0.002).  The isolated key question (irregular breathing) was successful and resulted in the additional capture of 22 OHCA patients in the new 12-D-3 irregular breathing determinant code. The decrease in OHCAs in target 12-A-1 appears to be linked to increase in OHCAs found in 12-D-3. | Advanced Medical Priority Dispatch System | One year difference in data collection periods. Possible confounders not considered. | Moderate |
| **Quantitative non-randomised study** |  | | | | | | |
| **Roppolo (2009) Dispatcher assessments for agonal breathing improve detection of cardiac arrest^32^** | | | | | | | |
| **Study Design**  **Aim** | **Date of Data Collection** | | **Setting/**  **Participants** | **Main Findings** | **Dispatch Software** | **Limitations** | **Quality Grade**  MMAT 2018 |
| Prospective before and after study.  The specific aim of this study was to determine whether the new protocol increased the detection of agonal respirations by EMDs and thus the presence of cardiac arrest. | Unclear | | US.  The emergency medical dispatch system in Dallas. | 22 patients with agonal breathing detected in the 4 months after protocol implementation compared with 0 patients in the 8 months prior.  After introduction of the agonal breathing protocol, the percentage of patients who did not have EMD criteria for cardiac arrest, but actually were in cardiac arrest decreased from 28.0% (168/599) to 18.8% (68/362; p = 0.0012), a yield of an additional 100 patients over the 4-month follow-up period.  Survival to ED admission was similar between the two groups; p = 0.9979.  Presenting rhythm was ventricular fibrillation (VF) or pulseless electrical activity (PEA) in 85% of those identified as having agonal breaths versus 46% without them (asystole; p = 0.002).  Bystanders started CPR significantly more frequently after the new protocol was instituted (60.9% before vs. 71.5% afterward, p = 0.006).  Dispatchers found that asking the bystander to say “now” every time they witnessed the patient breath was most helpful in detecting when these respirations did indeed occur.  Dispatchers also felt that putting the phone next to the patient was not helpful.  Dispatchers reported the use of the 10 s interval was very sensitive in that all cases identified as having agonal respirations had frequencies much less than six per minute. | Emergency Medical Dispatch Programme developed by The Association of Public Safety Communications Officials. | Hawthorne effect.  Non randomised design.  Did not investigate those patients breathing ‘normally’ so unclear if these patients triaged correctly. | Moderate |
| Quantitative non-randomised study |  | | | | | | |
| **Lewis (2013) Dispatcher-Assisted Cardiopulmonary Resuscitation: Time to Identify Cardiac Arrest and Deliver Chest Compression Instructions^33^** | | | | | | | |
| **Study Design**  **Aim** | **Date of Data Collection** | | **Setting/**  **Participants** | **Main Findings** | **Dispatch software** | **Limitations** | **Quality Grade**  MMAT 2018 |
| Retrospective Cohort Study | 2011 | | United States  590 OHCA EMS Calls | EMDs recognised OHCA in 80% of cases.  Where the EMD could assess consciousness and breathing the OHCA was not recognised in 8% of cases.  EMDs are less likely to recognise OHCA where the arrest is witnessed.  The caller is more likely to give uncertain or contradictory information regarding consciousness in 54.3% of cases where the OHCA was not recognised compared to 22% of cases where the arrest was recognised.  The patient was more likely to be reported as breathing, or contradictory information given in 74.3% of unrecognised cases as opposed to 35.7% of recognised cases | Unclear | 19% of eligible calls were unavailable for analysis due to tech issues.  Assumptions were made regarding what the EMD was thinking based on the EMS call recording | Moderate |
| **Quantitative non-randomised study** |  | | | | | | |
| **Hardeland (2014) Comparison of Medical Priority Dispatch (MPD) and Criteria Based Dispatch (CBD) relating to cardiac arrest calls^34^** | | | | | | | |
| **Study Design**  **Aim** | **Date of Data Collection** | | **Setting/**  **Participants** | **Main Findings** | **Dispatch software** | **Limitations** | **Quality Grade**  MMAT 2018 |
| Observational Study.  The aim of this study was to compare two commonly used medical dispatch tools in handling cardiac arrest calls; Medical Priority Dispatch (MPD) used in Richmond, USA and Criteria Based Dispatch (CBD) used in Oslo and Akershus, Norway. | MPD Site: 1^st^ May 2010 – 30^th^ April 2011  CBD Site: 1^st^ Jan 2007 – 31^st^ Dec 2007 | | Oslo & Akershus, Norway. Richmond, US. | Protocol adherence was similar for the MPD and CBD systems with 100 (100%) vs. 136 (97%) calls successfully clarifying consciousness (p = 0.14) and 100 (100%) vs. 137 (98%) clarifying respiratory arrest(p = 0.27), respectively. Absence of normal breathing was initially clarified by the dispatcher in 28 (28%) in the MPD system and 51 (36%) of the calls in the CBD system (p = 0.17), and cardiac arrest recognised in 82 (82%) and 108 (77%) (p = 0.42) calls respectively. In both systems the most frequent reason for not recognising cardiac arrest was misinterpretation of agonal breathing.  Pre-arrival CPR instructions were offered in 81% vs. 74% (p = 0.22) of eligible cases and declined in 7% vs. 5% (p = 0.58) in the MPD vs. CBD systems, respectively. The most frequent reason for not offering CPR instructions was failure to recognise cardiac arrest due to agonal breathing.  Pre-arrival CPR instructions were offered faster and more frequently in the CBD system, but in both systems chest compressions were delayed 3–4 min. Earlier recognition of cardiac arrest and improved CPR instructions may facilitate earlier lay rescuer CPR. | Medical Priority Dispatch and Criteria-Based Dispatch | Different years of data collection (2007 CBD site and 2010/11 MPD site)  may have confounded.  Small sample size. | Moderate |
| **Quantitative descriptive study** |  | | | | | | |
| **Travers (2014) Out-of-hospital cardiac arrest phone detection: Those who most need chest compressions are the most difficult to recognise^35^** | | | | | | | |
| **Study Design**  **Aim** | **Date** | | **Setting**  **Participants** | **Main Findings** |  | **Limitations** | **Quality Grade**  MMAT 2018 |
| Prospective Observational Study.  Aimed to measure prospectively the rate of OHCA recognition. | May 2012 | | France.  Paris Firefighter (FF) Dispatch Centre. | Dispatcher recognised 50/82 (61%) OHCA.  The comparison between detected and undetected OHCAs highlighted differences in assessment of ventilation status and victim outcome. The presence of agonal breathing and the absence or incomplete ventilation status assessment decreased the likelihood of recognizing OHCA.  There was a link between the request to put a hand on the abdomen and correct CA recognition (p = 0.001).  The median time and the interquartile range from the call to CA  recognition was 2 min 23 s (1 min 51 s to 3 min 7 s). Among the 50  detected CAs, 27 received dispatcher-assisted CPR.  The median interval from the call beginning to the CPR initiation  was 3 min 37 s (2 min 57 s to 5 min). | Unclear | Small sample.  Risk of subjective selection. | Moderate |
| **Quantitative non-randomised study** |  | | | | | | |
| **Møller (2016) Recognition of out-of-hospital cardiac arrest by medical dispatchers in emergency medical dispatch centres in two countries^36^** | | | | | | | |
| **Study Design**  **Aim** | **Date of Data Collection** | | **Setting/**  **Participants** | **Main Findings** | **Dispatch Software** | **Limitations** | **Quality Grade**  MMAT |
| Observational Registry Study.  To analyse and compare the accuracy of OHCA recognition by medical dispatchers in two countries. | Jul 2013 – Dec 2013 | | Sweden and Denmark | Using data from OHCA registers and EMDC data: The sensitivities for recognition of cardiac arrest was 40.9% (95% CI: 37.1–44.7%) in the Capital Region of Denmark and 78.4% (95% CI: 73.2–83.0%) in Skåne Region in Sweden (p < 0.001).  When also adding in data from emergency call recordings: The sensitivities for OHCA recognition were 80.7% (95% CI: 77.7–84.3%)in Denmark and 86.0% (95% CI: 81.3–89.8%) (p = 0.06) in Sweden.  Consciousness and breathing were addressed in general and more frequently in the recognised OHCA groups in both regions.  In Denmark 48% of audio recordings had missing dispatch codes.  “Unclear problem” (17%) was the most frequent dispatch code for audited non-recognised OHCA and possible death (12%) for audited recognised OHCA in Denmark.  In Sweden breathing difficulties was the most frequent dispatch code in both groups of audited cases (23% recognised and 21% unrecognised).  The highest priority response was provided in all but one of the audited cases where OHCA was recognised and in 90% and 97% of the audited non-recognised calls in the Capital Region and the Skåne Region, respectively. | Criteria-Based Dispatch | Differences between countries in data registration practices.    Differences in the proportion of missing data and ability to link data.  Criteria Based Emergency Medical Dispatch Systems.  Results may not be generalisable to alternative dispatch systems. | Moderate |
| **Quantitative descriptive study** |  | | | | | | |
| **Biancardi (2017) Cardiac arrest recognition and telephone CPR by emergency medical dispatchers^37^** | | | | | | | |
| **Study Design**  **Aim** | **Date of Data Collection** | | **Setting/**  **Participants** | **Main Findings** | **Dispatch Software** | **Limitations** | **Quality Grade**  MMAT 2018 |
| Simulation Study  To explore  the recognition of OHCA by EMDS and the frequency of telephone assisted CPR offered to  bystanders. | Unclear | | Malta  52 ED Nurses | In a simulation where the patient is not breathing 92% recognised OHCA, TCPR started in 75% of cases. 88% dispatched code red response.  In the agonal simulation 42% recognised OHCA, TCPR started in 41%. 77% dispatched a code red response. | Unclear | Simulation study so may not reflect ‘real life’ scenario.  Hawthorne Effect. | Moderate |
| **Quantitative descriptive study** |  | | | | | | |
| **Mirhaghi (2017) Recognizing Sudden Cardiac Arrest May Require More Than Two Questions during Telephone Triage: Developing a Complementary Checklist^38^** | | | | | | | |
| **Study Design**  **Aim** | **Date of Data Collection** | | **Setting/**  **Participants** | **Main Findings** | **Dispatch Software** | **Limitations** | **Quality Grade**  MMAT 2018 |
| Content analysis OHCA emergency calls.  To develop decision-support tools to identify patients experiencing sudden OHCA.  Simulated case scoring. | April 2015 – Jun 2015 | | Iran.  Emergency  Medical Dispatch Centre in Mashhad University  of Medical Sciences. | Content analysis of calls identified 3 main themes:  callers’ tone and presence of background voices, calling for ambulance and providing an address, and description of the patient’s  primary complaint and respiration status.  The mean (SD) percentages of correct responses was 66.9%±27.96% prior to the use of checklist and 80.05%±10.84% afterwards).  Dispatchers had correctly identified 68% and 66% of SCA and non-SCA cases; the use of the checklist increased this accuracy to 84% and 76%, respectively. | Unclear | Unclear how many dispatchers were invited to participate to evaluate selection bias.  Dispatchers used the checklist to triage calls they had already triaged, may have positively impacted scoring.  Simulation study, results may be different in practice. | Moderate |
| **Quantitative non-randomised study** |  | | | | | | |
| **Hardeland (2017) Targeted simulation and education to improve cardiac arrest recognition and telephone assisted CPR in an emergency medical communication centre^55^** | | | | | | | |
| **Study Design**  **Aim** | **Date of data collection** | | **Setting/**  **Participants**  **Time of data collection** | **Main Findings** | **Dispatch Software** | **Limitations** | **Quality Grade**  MMAT 2018 |
| Prospective Interventional Study  To evaluate the effectiveness of performance-based education, training and feedback on cardiac arrest call handling in Oslo University Hospital EMCC | 2014 | | Norway  All OHCA calls  Pre-intervention Jan 2013 – Jan 31^st^ 2014  Post-intervention May 14^th^ 2014 – Dec 31^st^ 2014 | Significant improvement in recognition of OHCA following the intervention (89 vs. 95%, p = 0.024).  Delayed recognition significantly reduced following the intervention (21 vs. 6%, p < 0.001). | Criteria-Based Dispatch | Risk of confounding. Non-randomised design. Differing time periods before/after intervention. | Moderate |
| **Quantitative descriptive study** |  | | | | | | |
| **Riou (2018) ‘She’s sort of breathing’: What linguistic factors determine call-taker recognition of agonal breathing in emergency calls for cardiac arrest?^39^** | | | | | | | |
| **Study Design**  **Aim** | **Date of Data Collection** | | **Setting/**  **Participants** | **Main Findings** | **Dispatch Software** | **Limitations** | **Quality Grade**  MMAT 2018 |
| Retrospective Linguistic Analysis.  Investigating recognised and unrecognised emergency calls for confirmed OHCA.  To explore whether the language used by callers to describe breathing impacts on call-taker  recognition of agonal breathing and hence cardiac arrest. | Jan 2014 – Dec 2015 | | St Johns Ambulance Western, Perth, Australia.  176 emergency calls of paramedic confirmed OHCA.  MPDS Dispatch Software. | Amongst recognised and unrecognised calls, the breathing status question received an answer in 89% of calls. 64% initially reported as breathing.  OHCA recognised in 28% of calls with a yes answer, 95% with a no answer and 79% with a non-answer response.  32% of answers to the breathing question were qualified.  Qualification found in 44% of yes answers. Qualified yes answers were suggestive of agonal breathing, but they were treated similarly to plain yes answers.  Call-takers entered in ProQA that the patient was breathing after  94% (47/50) of qualified yes-answers and 94% (59/63) of plain yes answers.  OHCA was subsequently recognised in 22% (14/63) of calls with a plain yes-answer and in 36% (18/50) of calls with a qualified yes-answer. The odds of OHCA recognition were not significantly higher following a qualified yes-answer rather than a plain yes-answer (Odds Ratio 1.96; 95% Confidence Interval 0.86-4.57; p = 0.11).  Overall, the median time to the breathing sequence was 56 s (IQR 44–72) from call start, and the median duration of the breathing sequence was 5 s (IQR 3–10). The median duration of the breathing sequence was significantly shorter (p < 0.001) in calls where OHCA was not recognised (median 4 s, IQR 3–7) than in calls where OHCA was recognised (median 7 s, IQR 4–12). | Medical Priority Dispatch Software V12.1.3, implemented with ProQA software. | Small sample of 176 calls.  As the calls were stratified for OHCA recognition,  our results are not representative of the whole population of OHCA  cases.  MPDS dispatch system and may not be generalisable beyond this system. | Moderate |
| **Quantitative non-randomised study** |  | | | | | | |
| **Derkenne (2020) Improving Emergency Call Detection of Out-of-Hospital Cardiac Arrests in the Greater Paris Area: Efficiency of a Global System with a New Method of Detection^40^** | | | | | | | |
| **Study Design**  **Aim** | **Date** | | **Setting/**  **Participants**  **Time of data collection** | **Main Findings** | **Dispatch Software** | **Limitations** | **Quality Grade**  MMAT 2018 |
| Repeated cross-sectional design to assess performance improvement in a Dispatcher Assisted CPR programme (DA-CPR) over the period of 2012 to 2018. | 2012-2018 | | Paris, France.  OHCA patients cared for by a Basic Life Support Team during the study period. | Dispatchers correctly identified 54% of recognizable OHCAs in 2012, 76% in 2015, 83% in 2017, and 93% in 2018.  In 2012, dispatchers assessed breathing for 71% of patients, and this proportion increased to 97% in 2018.  t-CPR performance increased from 51% to 84% during the 6 years of observations.  The rate of dispatchers searching for a defibrillator increased from 0 to 20% and the rate of ongoing CC at BLS arrival increased from 36% to 83% (p = 0.01).  In contrast, the rate of CPR-bystander before call did not improve significantly (4% to 17%, p = 0.22), nor did the survival rate.  After adjusting for confounders, OHCA detection was associated with breathing assessments, particularly when assessed with HoB (aOR: 13.1 95%CI: 4.8-39.5), during the 2018 period (aOR: 3.4, 95% CI: 1.1-10.8), and when the OHCA occurred in a public place (aOR: 0.14, 95%CI: 0.05-0.4), (compared to an OHCA at home).  Neither age nor the seniority of dispatchers was associated with the success of OHCA detection or t-CPR.  The sensitivity of HoB for CA detection was measured among patients at 96.2%.  Public places were adversely associated with the detection of OHCAs. | Unclear | Potential for confounders.  Different number of patients between each study period.  Questionable whether results are generalisable outside of the study context. | Moderate |
| **Quantitative non-randomised study** |  | | | | | | |
| **Mao (2020) Is your unconscious patient in cardiac arrest? A new protocol for telephonic diagnosis by emergency medical call-takers: A national study^41^** | | | | | | | |
| **Study Design**  **Aim** | **Date of Data Collection** | | **Setting/**  **Participants** | **Main Findings** | **Dispatch Software** | **Limitations** | **Quality Grade** |
| Prospective before and after study  Sought to determine the sensitivity,  specificity, likelihood ratios, time to diagnosis and time to 1^st^ compression when the modified protocol was introduced for unconscious patients. | July 2018 | | Singapore.  513 EMS calls for unconscious patients | Only a 50.4% adherence to the ‘after’ protocol which included assessing breathing using hand on belly technique.  Analysed on intention to treat basis and ‘after’ group had a diagnostic accuracy of 84.4% opposed to 67.5% in the ’before’ group.  No significant change in time to chest compressions.  Standard breathing question is problematic. | Unknown | Low adherence to the ‘after protocol’.  Risk of bias – test-retest bias, observer bias, history bias. | Moderate |
| **Quantitative non-randomised study** |  | | | | | | |
| **Schwarzkoph (2020) Seizure-like presentation in OHCA creates barriers to dispatch recognition of cardiac arrest^42^** | | | | | | | |
| **Study Design**  **Aim** | **Date of Data Collection** | | **Setting/**  **Participants** | **Main Findings** | **Dispatch Software** | **Limitations** | **Quality Grade** |
| Retrospective cohort study | 2014-2018/2020 | | United States  3502 OHCA EMS calls | In the seizure activity group there were significant delays in the EMD asking consciousness and breathing questions and establishing abnormalities.  The seizure activity group had a longer median time to the EMD establishing OHCA.  The seizure activity group were often described as abnormal breathing and turning blue, purple or red. | Unclear | 4 year study period and confounders not considered.  Comparatively small number where seizure activity described compared with non-seizure activity (149v3353) | Moderate |
| **Quantitative descriptive study** |  | | | | | | |
| **Stangenes (2020) Delays in recognition of the need for telephone-assisted CPR due to caller descriptions of chief complaint^43^** | | | | | | | |
| **Study Design**  **Aim** | **Date of Data Collection** | | **Setting/**  **Participants** | **Main Findings** | **Dispatch Software** | **Limitations** | **Quality Grade** |
| EMS call analysis | Unclear/2020 | | United States  434 OHCA EMS calls | The way the caller describes the chief complaint effects OHCA recognition and delays tCPR delivery with the greatest delay for incorrect medical condition complaints.  EMDs often pursue questioning relating to the specific diagnostic condition at the expense of key consciousness and breathing questions. | Criteria-Based Dispatch | Unclear how real cardiac arrest calls were selected.  Data was part of a randomised controlled simulation study and may not be generalisable to other systems.  The results may not be generalisable to EMS systems not using Criteria-Based Dispatch | Moderate |
| **Quantitative descriptive study** |  | | | | | | |
| **Tamminen (2020) Spontaneous trigger words associated with confirmed out-of-hospital cardiac arrest: a descriptive pilot study of emergency calls^44^** | | | | | | | |
| **Study Design**  **Aim** | **Date of Data Collection** | | **Setting/**  **Participants** | **Main Findings** | **Dispatch Software** | **Limitations** | **Quality Grade** |
| Descriptive pilot study - retrospective registry study analysing linguistic content.  to examine the association between true OHCA confirmed by  ambulance personnel and laypeople’s spontaneous trigger words regarding physiological deterioration of a patient in the context of emergency-dispatcher-suspected or EMS encountered OHCA. | Jan 2017 – May 2017 | | Finland.  80 emergency  calls of dispatcher suspected or EMS encountered OHCA. | 64% of the sample were confirmed as true cardiac arrests, and 36% were regarded as non-cardiac arrest events. Most cardiac arrests were suspected after an ambulance was dispatched, and two confirmed cardiac arrests were not recognised by the dispatcher. A total of 291 spontaneous trigger words were analysed; 32% and 14% of them concerned breathing and altered level of consciousness, respectively.  Spontaneous trigger words that were more frequently used to describe true cardiac arrest were ‘is not breathing’ (n = 9 [18%], ‘the patient is blue’ (n = 9 [18%], ‘collapsed or fallen down’ (n = 12 [24%], and ‘is wheezing’ (n = 17 [33%], ‘Is snoring’ was associated with a false suspicion of cardiac arrest (n = 1 [2%] vs n = 6 [21%]. | Unclear | Underpowered to show association.  Uncertain if generalisable outside of Finland. | Moderate |
| **Quantitative non-randomised study** | |  | | | | | |
| **Gram (2021) Assessment of a quality improvement programme to improve telephone dispatchers’ accuracy in identifying out-of-hospital cardiac arrest^45^** | | | | | | | |
| **Study Design**  **Aim** | **Date of Data Collection** | | **Setting/**  **Participants** | **Main Findings** | **Dispatch Software** | **Limitations** | **Quality Grade** |
| Quality assessment study.  To analyse and compare the accuracy of the Emergency Medical Dispatch Centre in identifying OHCA before and after an educational intervention | 2017-2020 | | Denmark  673 OHCA EMS calls | Significant difference in the sensitivity of identifying OHCA between pre-intervention and post-intervention periods.  Sensitivity pre-intervention 82.3% (95% CI: 76.4-87.2%)  Sensitivity post-intervention 92.7% (95% CI: 88.2-95.8%)  (p=0.0014)  Time to answer the key questions did not improve. | Unclear | Confounding factors not considered | Moderate |
| **Quantitative non-randomised study** |  | | | | | | |
| **Riou (2021) ‘I think he’s dead’: A cohort study of the impact of caller declarations of death during the emergency call on bystander CPR^56^** | | | | | | | |
| **Study Design**  **Aim** | **Date of Data Collection** | | **Setting/**  **Participants** | **Main Findings** | **Dispatch Software** | **Limitations** | **Quality Grade** |
| To examine the relationship between caller’s declarations of death and their response to dispatcher’s initiation of CPR and to OHCA recognition | 2014-2015 | | Australia  Non-traumatic adult OHCA | There was a significant difference between declarations of death and whether the OHCA was witnessed, or unwitnessed. Callers were more likely to make a declaration of death in an unwitnessed event.  Initial recognition of OHCA was significantly more frequent where the caller made a declaration of death.  A caller was significantly more likely to decline to do CPR where they had made a declaration of death before dispatch.  In 15% of cases where callers made a declaration of death achieved prehospital ROSC and 9% had ROSC on arrival at hospital. | Advanced Medical Priority Dispatch System | The study examined initial OHCA recognition, but only included those that were recognised by the EMD. The study excluded those patients that continued to be unrecognised and that were also not resuscitated by EMS. | Moderate |

Supplementary Table S5

*Quantitative Papers (Grouped by certainty) and listed in descending order of date of publication*

**Low Certainty Quantitative Papers**

| **Quantitative Descriptive Studies** |  | | | | | |
| --- | --- | --- | --- | --- | --- | --- |
| **Bang (2003) Interaction between emergency medical dispatcher and caller in suspected out-of-hospital cardiac arrest calls with focus on agonal breathing. A review of 100 tape recordings of true cardiac arrest cases^46^** | | | | | | |
| **Study Design**  **Aim** | **Date of data Collection** | **Setting/**  **Participants** | **Main Findings** | **Dispatch Software** | **Limitations** | **Quality Grade**  MMAT 2018 |
| Prospective study evaluating 100 tape recordings of EMS calls.  To assess EMD ability to identify and prioritise OHCA and offer CPR.  To assess frequency and caller’s descriptions of agonal breathing. | Sep 2000-Oct 2001 | Sweden  EMS calls for OHCA recorded at 1 dispatch centre. | Level of Consciousness only questioned in 75% of cases.  Respiration only questioned in 2/3 of cases. Is s/he breathing normally? Only raised in 41% of cases.  In 2/3 of cases the quality of the interview was highly commended, 11% insufficient, 26% unapproved – important questions omitted.  There was an opportunity to identify 79% of cases, but only 62% identified.  69% callers were calm, 96% cooperative.  16% of patients were reported awake at start of call.  2/3 patients it was clear that unconscious and not breathing/normally.  In 10% cases no ALS dispatched despite signs of a life-threatening condition. | Unclear | No detail on how the 100 calls were selected.  Only included OHCA patients admitted to hospital.  Deceased on scene omitted from study. | Low |
| **Bohm (2009) Tuition of emergency medical dispatchers in the recognition of agonal respiration increases the use of telephone assisted CPR^47^** | | | | | | |
| **Study Design**  **Aim** | **Date of Data Collection** | **Setting/**  **Participants** | **Main Findings** | **Dispatch Software** | **Limitations** | **Quality Grade**  MMAT 2018 |
| Before and after study.  Investigating whether tuition in recognising agonal breathing improves EMD identification of OHCA and the offer of TCPR. | 2004-2006 | Stockholm, Sweden. | There was a high willingness to receive CPR instructions among the bystanders, 97% in 2004 and 100% in 2006.  T-CPR was offered in 47% (n=36) of cases before tuition and 68%(n=52) after (p=0.01).  In agonal respiration 23% had been offered T-CPR in 2004 whereas 56% were offered T-CPR in 2006(p=0.006).  After tuition, there were at least five instances when the EMD did not offer T-CPR. The main reason for the EMD not to recognize CA and failing to offer T-CPR in these cases was their inability to recognize abnormal/agonal respiration. | Medical Index Protocol | The months of the year are different in the 2 separate sampling periods. Limited mention of potential confounders. | Low |

Supplementary Table S6

*Qualitative Papers (Grouped by quality)*

**High Certainty Papers**

| **Qualitative Studies**  **HIGH QUALITY** |  | | | | | |
| --- | --- | --- | --- | --- | --- | --- |
| **Bang (2002) Dispatcher-assisted telephone CPR: a qualitative study exploring how dispatchers perceive their experiences^48^** | | | | | | |
| **Study Design**  **Aim** | **Date of Data Collection** | **Setting/**  **Participants** | **Main Findings** | **Dispatch Software** | **Limitations** | **Quality Grade** |
| Qualitative semi-Structured Interview Study.  To assess the emergency medical dispatchers’ ability to identify and prioritise OHCA and offer T-CPR and to give an account of the frequency of agonal respiration and the  caller’s descriptions of breathing. | Unclear | Sweden  10 Emergency Medical Dispatch Staff | 12 categories and 31 subcategories.  Categories for perception in identifying OHCA were; trust the witness’s account, be open-minded and to be organised.  Categories for perception in offering t-CPR were: to feel prepared to connect with the witness on a mental level by being organised, flexible and supportive, to obtain a basis for assessments and to be observant for diverse obstacles in a situation.  Categories for perception in providing t-CPR were: to feel engaged, to be supportive of the witness, to feel secure by recognising response-feedback from the witness, to observe external conditions with regard to the locality and technical complications, to be composed and adjust to the needs of the situation, to feel competent or to feel despair. | Unclear | Small sample  Possibility of selection bias. | High |
| **Riou (2018) Hijacking the dispatch protocol: When callers pre-empt their reason-for-the-call in emergency calls about cardiac arrest^49^** | | | | | | |
| **Study Design** | **Date of Data Collection** | **Setting/**  **Participants** | **Main Findings** | **Dispatch Software** | **Limitations** | **Quality Grade** |
| Conversation analysis of a subset of 66 OHCA emergency calls where the caller pre-empted a reason for the call.  To explore trajectories of emergency calls where the caller pre-empted a reason for the call. | 2014/2015 | Western Australia.  A subset of 66/200 OHCA emergency calls where the caller pre-empted a reason for the call. | Reason-for-the-call pre-emption is a common practice in emergency calls processed with a dispatch protocol in which the address and telephone number are the first two orders of business.  Caller pre-emptions typically occur very early in the call, often as early as the caller’s first turn. They often receive minimum tokens of receipt by call-takers but are not explicitly attended to as they arise.  The main challenge that pre-emptions pose for call-takers is when they open the “official” reason-for the-call sequence later in the calls. If call-takers deliver the scripted turn “okay tell me exactly what happened” as usual, callers tend to treat it as a request for more information. They rarely repeat the reason-for-the-call they already pre-empted, and they can occasion delays by providing additional, superfluous information.  Out of the 66/200 callers who had pre-empted a reason-for-the-call earlier on, 10 callers (15%) prefaced their response to the official prompt (“okay tell me exactly what happened”) with “I don’t know” or “dunno”. By contrast, in the 134/200 calls without pre-emption, only 6 callers (4%) responded to “okay tell me exactly what happened” with an I-don’t-know preface. The difference was found to be statistically significant (χ2, p=0.02). | Advanced Medical Priority Dispatch System | No discussion of reflexivity.  No clear detail of the conversation analysis methodology. | High |

Supplementary Table S7

*Qualitative Papers (Grouped by quality)*

**Moderate Certainty Papers**

| **Qualitative Studies**  **MODERATE QUALITY** |  | | | | | |
| --- | --- | --- | --- | --- | --- | --- |
| **Jensen (2012)** **Factors associated with the successful recognition of agonal breathing and cardiac arrest by 9-1-1 communications officers: A qualitative iterative survey^50^** | | | | | | |
| **Study Design**  **Aim** | **Date of Data Collection** | **Setting/**  **Participants** | **Main Findings** | **Dispatch Software** | **Limitations** | **Quality Grade** |
| Qualitative Telephone Interview Study using the Theory of Planned Behaviour.  To identify and describe barriers and facilitators perceived to influence the recognition of abnormal breathing and delivery of T-CPR. | 2009 | Canada.  24 Ambulance Communication Officers (ACOs). | Retaining the top 75% of themes for each construct resulted in six behavioural, seven subjective normative, and 13 control beliefs. Control beliefs appeared to have the most important influence on the intention of ACOs to recognise abnormal breathing and administer CPR instructions. Subjective norms played a minor role. | Advanced Medical Priority Dispatch System | No discussion of reflexivity.  Unable to identify origin of quotes from supplemental data. | Moderate |
| **Alfsen (2015) Barriers to recognition of out-of-hospital cardiac arrest during emergency medical calls: a qualitative inductive thematic analysis^51^** | | | | | | |
| **Study Design**  **Aim** | **Date of data Collection** | **Setting/**  **Participants** | **Main Findings** | **Dispatch Software** | **Limitations** | **Quality Grade** |
| Inductive Thematic Analysis OHCA emergency calls.  To identify factors affecting medical dispatchers’ recognition of OHCA during emergency calls in a qualitative analysis of calls. | 2012 | Denmark  13 emergency calls of unrecognised OHCA and 8 emergency calls of recognised OHCA. | 3 themes: Caller’s physical distance, caller’s emotional distance, caller is a healthcare professional.  Caller’s physical distance (caller near patient, caller not near patient, caller assesses the patient).  Emotional distance (keeping calm, losing control).  Caller is a healthcare professional (responsibility is handed over to the caller, caller assumes responsibility). | Criteria-Based Dispatch – nationwide priority tool | No discussion of reflexivity.  Minimal detail regarding how themes were agreed. | Moderate |

Supplementary Table S8

*Mixed methods paper*

**High Certainty Paper**

| Mixed Methods Study  HIGH QUALITY |  | | | | | |
| --- | --- | --- | --- | --- | --- | --- |
| **Hardeland (2016) Factors impacting upon timely and adequate allocation of prehospital medical assistance and resources to cardiac arrest patients^52^** | | | | | | |
| **Study Design**  **Aim** | **Date of data Collection** | **Setting/**  **Participants** | **Main Findings** | **Dispatch Software** | **Limitations** | **Quality Grade**  MMAT 2018 |
| Observational data, non-participant observation and in-depth interviews.  To explore, understand and address issues that impact upon timely and adequate allocation of prehospital medical assistance and resources to out-of-hospital cardiac arrest patients. | 2013/2014 | Norway  Data from 3 x Norwegian EMC Centres and 19 interviews with dispatchers. | There were significant site differences in their adherence to algorithm (clarification of consciousness and normal breathing) (90, 96 and 72%,  respectively, p < 0.001), recognition of cardiac arrest (89, 94 and 78%, respectively, p < 0.001) and provision of CPR instructions (83, 83 and 61%, respectively, p < 0.001). The most frequent reason for delayed or failed recognition of cardiac arrest was misinterpretation of agonal breathing.  Qualitative themes were:  Protocol use and platform of knowledge,  Situational assessment,  Interrogation strategy/Assessment of breathing. | Criteria-Based Dispatch – Norwegian Index for Emergency Medical Assistance | Based on Criteria Based Dispatch and may not be generalisable to other dispatch systems. | High |

Supplementary Table S9

*Mixed methods paper*

**Moderate Certainty Paper**

| Mixed Methods Study  MODERATE QUALITY |  | | | | | |
| --- | --- | --- | --- | --- | --- | --- |
| **Watkins (2021) Predictors of recognition of out of hospital cardiac arrest by emergency medical services call handlers in England: a mixed methods diagnostic accuracy study^53^** | | | | | | |
| **Study Design**  **Aim** | **Date of data Collection** | **Setting/**  **Participants** | **Main Findings** | **Dispatch Software** | **Limitations** | **Quality Grade**  MMAT 2018 |
| Mixed methods retrospective study using qualitative call analysis and data analysis.  To identify key indicator symptoms and patient factors associated with correct OHCA dispatch allocation. | 2013-2014 | United Kingdom | Key indicator symptoms for OHCA were ‘not breathing’. Reports of effective breathing, abnormal pulse, or heart rate, fluctuating level of consciousness and being female decreased the likelihood of OHCA being identified.  Estimated sensitivity of 72.8% (CI 65.8 to 79.1%) and a specificity of 99.4% (CI 99.3 to 99.6%) for call handlers’ recognition of OHCA.  Complete adherence to the dispatch protocol would have increased sensitivity by 7%, but reduced specificity to 95.7%. | Advanced Medical Priority Dispatch System | Risk of selection bias as patients who were not conveyed to hospital were excluded.  Small sample sizes.  Findings may not be generalisable to EMS services not using AMPDS. | Moderate |
